# Contrast gain control in mouse auditory cortex

**DOI:** 10.1152/jn.00847.2017

**Published:** 2018-07-25

**Authors:** James E. Cooke, Andrew J. King, Ben D. B. Willmore, Jan W. H. Schnupp

**Affiliations:** ^1^Department of Physiology, Anatomy and Genetics, University of Oxford, Oxford, United Kingdom; ^2^University College London, United Kingdom; ^3^Department of Biomedical Sciences, City University of Hong Kong, Hong Kong

**Keywords:** auditory cortex, contrast, gain control, mouse, spectrotemporal receptive field

## Abstract

The neocortex is thought to employ a number of canonical computations, but little is known about whether these computations rely on shared mechanisms across different neural populations. In recent years, the mouse has emerged as a powerful model organism for the dissection of the circuits and mechanisms underlying various aspects of neural processing and therefore provides an important avenue for research into putative canonical computations. One such computation is contrast gain control, the systematic adjustment of neural gain in accordance with the contrast of sensory input, which helps to construct neural representations that are robust to the presence of background stimuli. Here, we characterized contrast gain control in the mouse auditory cortex. We performed laminar extracellular recordings in the auditory cortex of the anesthetized mouse while varying the contrast of the sensory input. We observed that an increase in stimulus contrast resulted in a compensatory reduction in the gain of neural responses, leading to representations in the mouse auditory cortex that are largely contrast invariant. Contrast gain control was present in all cortical layers but was found to be strongest in deep layers, indicating that intracortical mechanisms may contribute to these gain changes. These results lay a foundation for investigations into the mechanisms underlying contrast adaptation in the mouse auditory cortex.

**NEW & NOTEWORTHY** We investigated whether contrast gain control, the systematic reduction in neural gain in response to an increase in sensory contrast, exists in the mouse auditory cortex. We performed extracellular recordings in the mouse auditory cortex while presenting sensory stimuli with varying contrasts and found this form of processing was widespread. This finding provides evidence that contrast gain control may represent a canonical cortical computation and lays a foundation for investigations into the underlying mechanisms.

## INTRODUCTION

Understanding the brain as an information processing system requires an understanding of the computations performed by different neural populations and their mechanistic underpinnings (Poggio 2012). It has been argued that certain computations are “canonical” in the sense that they are employed by different populations of neurons in different areas of the brain and in different species (Carandini and Heeger 2012). Contrast gain control (CGC), a compensatory reduction in the gain of sensory evoked activity in response to increased stimulus contrast, may represent such a computation. CGC has been observed in individual neurons in different sensory systems and in a variety of species, including mice (Atallah et al. 2012), rats (Garcia-Lazaro et al. 2007), rabbits (Brown and Masland 2001), guinea pig (Manookin and Demb 2006; Zaghloul et al. 2005), cats (Heeger 1992; Shapley and Victor 1978), ferrets (Rabinowitz et al. 2011, 2012), monkeys (Benardete et al. 1992; Carandini and Heeger 1994), and humans (Busse et al. 2009), as well as in nonmammalian species such as songbirds (Baccus 2006; Nagel and Doupe 2006), salamanders (Baccus and Meister 2002; Kim and Rieke 2001; Smirnakis et al. 1997), and bullfrogs (Rieke et al. 1995). In the mammalian cortex, it been observed in sensory areas, including primary visual (V1) (Carandini et al. 1997; Carandini and Heeger 1994; Heeger 1992; Heeger et al. 1996) and auditory (A1) (Rabinowitz et al. 2011) and somatosensory cortex (S1) (Garcia-Lazaro et al. 2007), as well as in higher visual cortical areas MT (Heeger et al. 1996; Rust et al. 2005; Simoncelli and Heeger 1998), V4 (Reynolds and Desimone 2003), and inferior temporal cortex (IT) (Zoccolan et al. 2005). The relative reward value of regions of visual space has also been found to induce divisive gain control in visual neurons in parietal cortex (Louie et al. 2011) as has visuospatial attention in a variety of visual cortical areas (Reynolds and Heeger 2009). The widespread nature of gain control supports the idea that it represents a canonical neural computation (Carandini and Heeger 2012).

The canonical nature of a computation does not necessarily imply that it is implemented by a common mechanism across modalities or brain structures (Carandini and Heeger 2012). Biophysical mechanisms for gain control have been identified in multiple animal models and these have been found to differ between mammalian and nonmammalian species (Olsen et al. 2010; Sadagopan and Ferster 2012). Within mammals, however, the cortex has long been thought to comprise canonical circuits that have evolved to implement equivalent computations across a variety of modalities (Douglas and Martin 1991), and so it is possible that, within the cortex, CGC may be implemented by a common mechanism, or combination of mechanisms, across sensory systems and species. In keeping with this, it is the computational relationship between stimulus contrast and spiking response gain that represents the canonical aspect of CGC, not the mechanism by which it is implemented.

To investigate the mechanisms underpinning computations in the mammalian brain, investigators are increasingly turning to the mouse as a model organism (Atallah et al. 2012; Olsen et al. 2012; Wilson et al. 2012; Wu et al. 2011). This is largely due to the abundance of genetic manipulations that are possible in this species (Akerboom et al. 2013; Boyden et al. 2005; Chow et al. 2010; Huang and Zeng 2013; Sternson and Roth 2014; Zhang et al. 2007) as well its suitability for other circuit-level techniques, such as head-fixed whole cell recording (Wu et al. 2011; Zhou et al. 2014), and two-photon imaging (Akerboom et al. 2013; Fu et al. 2014; Vasquez-Lopez et al. 2017). Exploiting these techniques to investigate CGC in the mouse auditory cortex should not only increase our understanding of the neural basis of this computation in the auditory system but may also inform the debate regarding the canonical nature of cortical circuitry (Douglas and Martin 1991). It is first necessary, however, to demonstrate experimentally that the mouse auditory cortex neurons actually exhibit CGC.

A valuable piece of information that can constrain circuit hypotheses is how the strength of CGC varies across cortical layers. If thalamocortical inputs drive CGC, gain changes should be observed in layer 4, where thalamic inputs are strongest. Gain changes may subsequently be inherited in other cortical layers, resulting in homogenous gain control across the depth of cortex. If layer-specific intracortical mechanisms underlie CGC, however, variation in the strength of CGC across different layers should be observed. Such an intracortical circuit for gain control has been identified in primary visual cortex (V1), where thalamic input drives corticothalamic projecting layer 6 neurons, which in turn drive a translaminar projecting parvalbumin (PV) interneuron subtype via facilitating synapses (Bortone et al. 2014; Olsen et al. 2012). Inhibition via this route has been found to reduce gain across all other cortical layers but not layer 6. If this circuit underlies CGC in auditory cortex, CGC should be absent from layer 6. Knowledge of the laminar distribution of CGC in the mouse visual and auditory cortex would serve to distinguish the likelihood of these different circuit hypotheses.

Here we characterized CGC in the mouse auditory cortex to lay a foundation for circuit-level dissection of the mechanisms involved in this computation. This line of investigation permits direct comparison with findings in visual and other sensory modalities, allowing the question of whether a canonical gain control mechanism exists to be addressed experimentally. Finally, we examined the laminar organization of CGC to constrain possible circuit hypotheses regarding the mechanistic basis of CGC in auditory cortex.

## MATERIALS AND METHODS

### 

#### Surgical methods.

We performed extracellular recordings from 815 sites in the left auditory cortex of 24 C57BL/6 mice. The animals were between 8 and 12 wk old at the time of recording. At this age, the range of cortical neuron best frequencies has been found to almost match that obtained for thalamocortical axon terminals in a C57BL/6 strain in which the Cdh23ahl allele that predisposes this strain to age-related high-frequency hearing loss has been corrected (Panniello et al. 2018; Vasquez-Lopez et al. 2017). It is therefore unlikely that the contrast gain control observed in this study would have been markedly affected by high-frequency hearing loss. All experiments were approved by the local ethical review committee and carried out under license from the UK Home Office in accordance with the Animal (Scientific Procedures) Act (1986). Recordings were performed while the animals were under ketamine-medetomidine anesthesia, using multichannel silicon probes (NeuroNexus, Ann Arbor, MI), while we presented auditory stimuli to the contralateral ear. Because the same animals were used for additional optogenetic experiments not described here, we collected data from transgenic mice expressing cre-recombinase under the PV promoter. One-third of the mice used (*n* = 8) were crosses between homozygous PV^cre^ (Jax No. 008069) (Hippenmeyer et al. 2005) and homozygous Ai35D mice (Jax No. 012735) (Madisen et al. 2012), which express ARCH and GFP in a cre-dependent manner. The other two-thirds (*n* = 16) were PV^cre^ mice that expressed channelrhodopsin (ChR2) and enhanced yellow fluorescent protein (EYFP) in PV interneurons. To achieve this expression, we performed intracranial injections of an adenoassociated virus (AAV-EF1a-DIO-hChR2(H134R)-EYFP-WPRE-pA serotype 2) (UNC Vector Core, Chapel Hill, NC) in the auditory cortex of these mice, several weeks bef ore recording experiments were performed. These genetic manipulations were designed to make PV+ interneurons in these mice light sensitive, but no optical stimulation was used in the experiments described here to characterize CGC in mouse cortex. We have verified that very similar results are obtained from the auditory cortex of wild-type C57BL/6 mice, and we note that the nature of the contrast gain control observed here is very similar to that previously reported for wild-type ferrets. The genetic background is therefore unlikely to be a factor in the experiments described below and is described here only for completeness.

The location of auditory cortex was identified using cranial landmarks. Craniotomies extended from the lambdoid suture at the most caudal extent to 1 mm rostral of the point at which the squamosal suture crosses the temporal ridge. In the dorsal-ventral axis, the craniotomy extended from 2 mm dorsal of the temporal line to the squamosal suture at the most ventral extent. Local vasculature was also used to localize the auditory cortex. Sites close to the largest vessel in this area oriented along the dorsal-ventral axis were consistently responsive to auditory stimuli. It was not possible to map-specific auditory cortical fields in these experiments and so auditory cortex recordings likely include responses from secondary as well as primary cortical areas. Frequency tuning of auditory responses was also used to confirm that recordings were localized to auditory cortex.

We recorded neural responses from all cortical layers simultaneously using single shank probes with 32 recording sites spaced 50 µm apart and arranged in a linear configuration (1 × 32). Compared with recordings made from ferret auditory cortex with these probes, spike amplitudes recorded from the mouse auditory cortex were relatively small, leading to fewer sites with robust responses than in the ferret. Traditional threshold-crossing methods for multiunit (MU) activity (MUA) extraction would have resulted in noisier estimates under these conditions. We therefore used an analog measure of MUA recorded on each channel instead. The analog MUA method used here provides high signal/noise estimates of neural activity [see Fig. 1 of Schnupp et al. (2015)]. It produces a measure of MUA measured in microvolts instead of spikes per second. It is not possible to recover reliable estimates of firing rates from threshold-crossing MUA methods, as such methods cannot distinguish large amplitude spikes from a collision of two or more smaller spikes, leading to an unpredictable and variable amount of “undercounting” of true spike events in threshold-based methods. In addition, threshold methods are also prone to overcounting when electrical noise generates false-positive threshold crossings. Methods that simply consider each spike as a wavelet and quantify stimulus-induced variation in signal energy in the frequency bands occupied by these wavelets (set here as 300–6,000 Hz) do not suffer from those sources of counting errors.”

This method has been used in several previous studies to extract MUA (Choi et al. 2010; Chung et al. 1987; Kayser et al. 2007; King and Carlile 1994; Schnupp et al. 2015; Schroeder et al. 1998). For each channel, we filtered the recorded voltage signal between 300 and 6,000 Hz. We then low-pass filtered the full-wave-rectified signal below 6,000 Hz and downsampled it to a sample rate of 12,000 Hz. We extracted local field potentials (LFPs) by low-pass filtering the recorded signals <300 Hz using a digital eighth order Chebyshev Type I filter.

#### Stimuli.

**Fig. 1.**
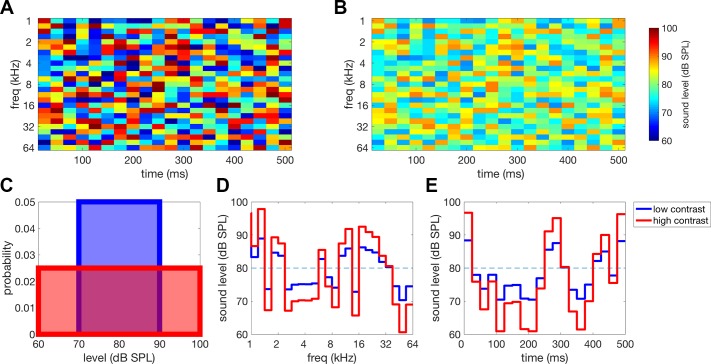
*A*: spectrogram of a high-contrast dynamic random chord (DRC) stimulus. DRCs are comprised of pure tones of specific frequencies that vary in their level over time, indicated by color on this plot. Levels for each tone at each time point are drawn randomly from a uniform distribution. For this high-contrast DRC, the range of possible intensities is ±20 dB. *B*: spectrogram of a low-contrast DRC. The range of possible intensities for this low-contrast DRC is ± 10 dB. *C*: distributions used to generate DRCs in *A* and *B*. The mean level for both distributions are the same, but the widths of the distributions, and therefore, the SDs are different. The increased width of the red distribution results in the generation of a higher contrast DRC (*A*) than that generated by the blue distribution (*B*). *D*: cross section though the DRC across frequencies for a given time point. At each time point, the width of the tone-level distribution also controls the variation in level across frequency. *E*: cross section though the DRC within a single frequency band. For a given frequency, the intensity fluctuates randomly over time. These fluctuations are larger for high-contrast DRCs generated using a tone-level distribution with a larger width (red). This increase in level variance results in an increase in the temporal contrast of the stimulus. This method of DRC generation leads to stimuli with matched spectral and temporal contrast.

We delivered dynamic random chord (DRC) stimuli to the animal during recording sessions. DRCs consist of a series of chords that themselves consist of a superposition of pure tones (Fig. 1, *A* and *B*). The frequencies of these tones are fixed, as is the duration of each chord. The intensity of each tone at any time point, however, is drawn from a distribution of possible values (Fig. 1*C*). This leads to fluctuations in intensity over both frequency (Fig. 1*D*) and time (Fig. 1*E*). The range of possible intensities therefore determines both the temporal and spectral contrast of the stimulus.

All DRCs in this study consist of 25 pure tone components at any one time, with frequencies log-spaced between 1 and 64 kHz, inclusive. The frequency range of the stimulus covered six octaves, and tone frequencies were spaced one-fourth octave apart. Chord duration was fixed at 25 ms, and 5-ms linear ramps were included between sequential chords to reduce “spectral splatter.”

To investigate CGC using these stimuli, the contrast of each DRC had to be controlled. The standard deviation of the stimulus sound level, and therefore the contrast, can simply be controlled by changing the range of tone intensities that comprise the DRC (Fig. 1, *B* and *C*). This method of controlling contrast changes both the variance in intensity across frequency bands (spectral contrast) (Fig. 1*D*) and the variance over time within frequency bands (temporal contrast) (Fig. 1*E*). We presented DRCs with one of two contrast levels.

In the low-contrast condition, the animal was presented with DRCs where the sound levels (SPL) of the tones were drawn from a uniform distribution with a mean of 80-dB SPL and a range of 20 dB (σLL = ~6.2 dB and c = σPP/µPP = 0.68). In the high-contrast condition, the tones had the same mean but the range was doubled to 40-dB SPL (σLL = ~11.97 dB and c = σPP/µPP = 1.2).

For these ranges, there is approximately a doubling in contrast from the low-contrast to high-contrast stimulus. Tone levels were generated using a variety of random seeds to generate different DRCs. Each DRC sequence consisted of 1,600 25-ms chords and lasted 40 s in total.

#### Stimulus presentation.

We presented stimuli using an Ultrasonic Dynamic Speaker (Avisoft Bioacoustics, Glienicke, Germany), modified for monaural in-ear delivery. The speaker was driven using a TDT RX6 multifunction processor at a sample rate of ~200 kHz. We calibrated stimuli across the frequency range of 1–64 kHz, using a Brüel & Kjær (Naerum, Denmark) Type 4138 1/8th-in. pressure-field microphone to assess the response of the speaker across this range. We then created an inverse filter based on this response, which was used to produce a flat frequency response in this frequency range, within ± 3 dB. We controlled stimulus presentation and data acquisition using in-house software (Benware; https://github.com/beniamino38/benware). Subsequent analysis was carried out in MATLAB (The MathWorks, Natick, MA).

#### Linear models.

DRCs have been used extensively to assess the spectrotemporal selectivity of auditory neurons (Ahrens et al. 2008; Bitterman et al. 2008; Christianson et al. 2008; deCharms et al. 1998; Linden et al. 2003; Rutkowski et al. 2002; Sahani and Linden 2003; Schnupp et al. 2001). Neurons in the auditory system typically respond to frequencies within a restricted range, as well as to temporal modulations, captured by the envelope of the sound. Spectrotemporal receptive fields (STRFs) are models of neuronal responses that are capable of capturing both of these features (Bizley et al. 2005; Kowalski et al. 1995; Shamma et al. 1993). Fluctuations in stimulus energy within frequency bands over time can be represented as the spectrogram of an auditory stimulus, *X(f,t)*. The STRF *k* is a linear filter that defines frequencies that drive or suppress neuronal responses over time, *yt*. The STRF captures spectral preferences of the neuron by assigning different weights to different frequency bins. At any given time in the stimulus, *t*, the STRF also weights the stimulus history, *h*, over a set number of time steps. We used sixteen 25-ms time steps here corresponding to 400 ms of stimulus history. The addition of this history dimension results in the stimulus being represented as a three-dimensional tensor, *Xtfh*. The STRF *kfh* captures features in the two dimensional frequency-history space that drive responses over time. Linear filtering of the stimulus *Xtfh*, by the STRF *kfh*, yields the predicted firing rate *ŷt*. The hat operator denotes an estimate of the parameter in question.

(1) $$\hat{y}t=k0+\overset{}{\underset{f,h}{\sum }}Xtfh.kfh$$

#### STRF estimation.

We excluded responses during the first second of auditory stimulation from the model fitting (to allow time for neurons to adapt to the initial contrast of the stimulus). We fitted linear STRFs and output nonlinearities to the remaining data by using gradient descent to minimize a sum-of-squares error term, *E*, that captures the degree to which the predicted MUA produced by the model *ŷt* differs from the actual MUA *yt*

(2) $$E=\overset{}{\underset{t}{\sum }}{\left(yt-\hat{y}t\right)}^{2}$$

We reduced the number of free parameters, and therefore the risk of overfitting, of the model by assuming that the tuning of the units is separable in both space and time (Ahrens et al. 2008). This involves fitting a frequency kernel, *k*f, and a history kernel, *k*_h_, and computing the outer product of these two kernels, *k*fh:

(3) $$k_{\text{fh}}\;=\;k_{\text{f}}⊗k_{\text{h}}$$

One limitation with this approach is that separable STRFs cannot model sensitivity to stimulus features that covary in frequency and time. This is compensated for, however, by the reduction in overfitting observed when using these models: separable STRFs tend to perform as well as or better than inseparable STRFs in predicting responses to unseen stimuli (Ahrens et al. 2008; Linden et al. 2003; Rabinowitz et al. 2011; Simon et al. 2007; Willmore et al. 2016). This advantage is especially pronounced when limited data are available on which to train the models due to the drastically smaller number of coefficients to be fitted for separable STRFs.

We fitted each kernel in turn by least-squares linear regression while the other kernel was fixed, and the process was repeated to convergence. Following fitting, we measured the best frequency (BF), spectral bandwidth and integration time of each STRF. The BF was defined as the frequency bin in the frequency kernel with the largest coefficient. The spectral bandwidth was measured as the width of the tuning curve around this peak response in the frequency kernel at half of its amplitude. For units that showed multiple peaks, the bandwidth was measured for the peak associated with the largest response. The temporal integration time was measured as the width of the tuning curve surrounding the peak coefficient observed in the first 100 ms of the history kernel. The measurement was taken at 50% of the amplitude of the peak coefficient.

#### Nonlinearities.

**Fig. 2.**
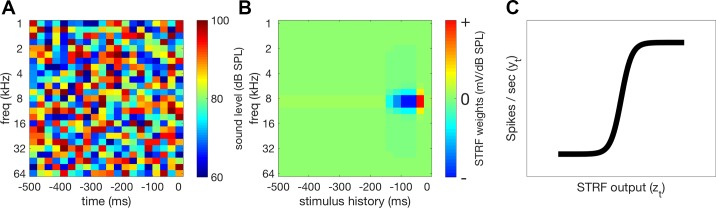
Model of neural responses. *A*: the aim of this model is to predict neuronal responses from the spectrogram of the DRC. *B*: the spectrogram is filtered through the Spectrotemporal receptive field (STRF) to calculate a predicted “neural activation function,” *z*t, which is linear with respect to the spectrogram. *C*: this linear activation is then passed through a sigmoidal output nonlinearity that maps STRF output *z*t onto the firing rate of the unit *y*t.

The linear STRF is capable of capturing important aspects of neuronal responses to auditory stimuli. However, the relationship between the spectrogram of a sound and neuronal responses is not truly linear, and it is therefore often advantageous to incorporate a nonlinearity into models to improve their accuracy. Here, we added a nonlinear output stage to the model, following the linear STRF. Instead of using the STRF to predict firing rates directly, the output of the STRF *z*t (Fig. 2) can be passed through a static sigmoidal output nonlinearity *F*, with parameters *a*, *b*, *c*, and *d* to be fit to the data (Fig. 2*C*):

(4) $$F\left[z\text{t}\right]=a+\frac{b}{1+e^{-\left(z\text{t}-c\right)d}}$$

This nonlinearity then maps STRF output, *z*t, onto predicted firing rate, *ŷt*:

(5) $$\hat{y}t\;=\;F\left[zt\right]$$

The shape of this nonlinearity results in rectification of the signal, preventing predictions of negative firing rates, and captures two major neuronal nonlinearities: response threshold and saturation. Adding this nonlinearity has been shown to improve predictions of neural responses in auditory cortex (Rabinowitz et al. 2011).

#### Current source density analysis.

We delivered bursts of broadband noise at 80-dB SPL lasting 50 ms in duration to the animal to assess whether neurons responded to auditory stimuli. In recordings made with linear probes, we used evoked local LFP responses to these noise bursts to identify cortical layers. The probe spanned all cortical layers and was inserted orthogonally to the cortical surface. We performed current source density (CSD) analysis on LFP responses to noise bursts to identify the pattern of current density flowing to and from the extracellular space, across depth and time. Current sinks are associated with synaptic activity, as this results in a net flow of current in to neurons and away from the extracellular medium, while current sources reflect the flow of current in to the extracellular medium (Nicholson and Freeman 1975; Pitts 1952). The pattern of current sinks and sources can be used to identify the location of electrode recording channels in different cortical layers.

Here, we used the inverse or iCSD method (Pettersen et al. 2006) to estimate the CSD for each channel, *Ĉz*. The aim of CSD analysis is to estimate the CSD from recorded potentials, Φ. The iCSD method solves this by first doing the inverse of this, calculating potentials for a known distribution of current sources. This method provides a matrix of the electrostatic forward solutions, *P*, that can be inverted to calculate current sources from recorded potentials:

(6) $$\hat{C}=P^{-1}\Phi$$

Several variants of the iCSD method exist; here we use the δ-source method. This method assumes that current sources originate from within infinitesimally thin disks around each electrode contact. If each disk is assumed to have an infinite radius, this method is equivalent to the standard method. Assuming a more plausible radius for each disk improves CSD calculations using this method. This radius corresponds to the area of cortex that is assumed to contribute to stimulus-evoked changes in current and voltage. Here we assumed a diameter of 250 µm. While assuming different values for this parameter can change CSD estimates, this has been found to not negatively impact on the use of CSDs for layer estimation (Szymanski et al. 2009).

The conductivity of the extracellular matrix must be factored into CSD calculations. Known anisotropic variation in extracellular conductivity can also be incorporated into this calculation, but we assumed a homogeneous, isotropic conductivity of 0.3 S/m. Changing the value of assumed extracellular conductivity does not alter the spatial or temporal pattern of CSDs used here for laminar identification, as it scales the CSD across all channels equally.

**Fig. 3.**
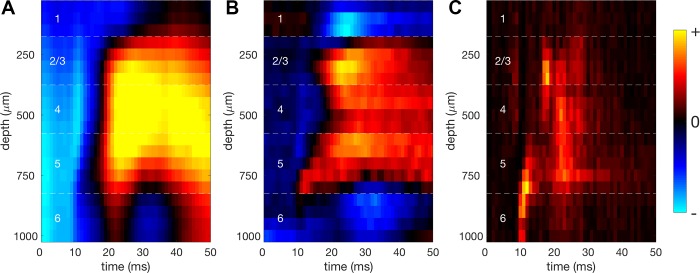
*A*: mean local field potential (LFP) responses to noise burst stimuli across 20 multielectrode channels on the linear probe, inserted orthogonally to the cortical surface. Channel 20 is deepest in the brain. All channels are separated by 50 µm. Since LFP signals are weakly localized in space, it is difficult to identify cortical layers from the LFP response. *B*: mean current source density responses to noise burst stimuli. The prominent reversal in polarity from channel 3 to channel 4 indicates the border of layer 1 and 2/3. All other layer boundaries (indicated by white broken lines) were assigned based on distance from this reference point. Distances between layer boundaries were obtained from published measurements of layer thickness (Anderson et al. 2009; Ji et al. 2016). White numbers indicate assignment of cortical layers. The early activity at the border of layer 2/3 and 4, as well as at the border of layer 5 and 6, corresponds to the known anatomy of thalamic input to the mouse auditory cortex. *C*: mean spiking responses to noise across all channels further validates layer assignment. Strong feedforward activity driven by thalamic input can be seen in layer 6 and lower layer 2/3. Spiking responses stop above layer 2/3, in keeping with the very sparse number of cell bodies found in layer 1. Warmer colors indicate increased activity (depolarization of LFP in *A*, current sinks in *B*, and an increase in firing rate in *C*) while colder colors indicate reduced activity (hyperpolarization of LFP in *A* and current sources in *B*; negative firing rates are not possible in *C*).

The most prominent feature of both the LFP and CSD is the reversal that occurs at the border of layers 1 and 2 (Fig. 3, *A* and *B*) (Christianson et al. 2011). This feature was used to align recordings so as to achieve consistent cortical depth measurements across animals. Layers were assigned based on experimentally obtained measurements of cortical thickness. While this will vary somewhat between animals, this variation has been found to be small relative to the spacing of our electrode sites (Anderson et al. 2009; Ji et al. 2016). Layers 2 and 3 were combined into a single layer 2/3, as is commonly done. Recordings between 0 and 225 µm below this border were classified as layer 2/3, between 225 and 425 µm as layer 4, between 425 and 675 µm as layer 5 and below 675 µm as layer 6. Layer assignments using this method were validated using CSD features, such as a prominent early sink corresponding to thalamic input in layer 6 and at the border of layers 2/3 and 4 (Fig. 3, *B* and *C*).

#### MU selection criteria.

We only submitted to further analysis recordings from penetrations in which MUA was responsive to noise burst stimuli, indicating that neurons in that area were acoustically driven. Furthermore, we required multiunits (MUs) to have STRFs that had at least some success at predicting neural responses to novel DRC stimuli. To quantify model performance, data from a single condition were divided into fitting and test sets. We fitted models to 90% of the data and then used the model to predict responses to the remaining 10% of the data. Performance was quantified as the correlation between predicted and actual responses in the test set. MU STRFs were deemed predictive if the correlation coefficient (CC) between the predicted and actual response on the test set was >0.04. This CC criterion was selected based on a statistical analysis in which we computed a null distribution of CCs from shuffled data (data not shown). We then chose a threshold criterion value that lay outside of this distribution of CCs that could be expected to occur by chance. MU STRFs that did not meet this criterion were excluded from subsequent analysis. We assessed cross-condition performance by training the model on the data from one condition and then using the model to predict responses to the stimulus from the other condition. We quantified performance using the correlation between the predicted and observed responses to the test stimuli.

## RESULTS

### 

#### Stimulus contrast has a suppressive effect on STRF tuning.

First, we assessed the effect of stimulus contrast on the tuning of auditory cortex neurons. In principle, changes in stimulus contrast might affect both neuronal tuning (which we measure here as the relative values of different coefficients in the STRF) and gain (the absolute values of the STRF coefficients). If contrast systematically alters tuning, then this would need to be taken into account when assessing gain changes. On the other hand, if contrast does not systematically affect tuning, then the relative values of the STRF coefficients should be largely constant across contrast conditions.

**Fig. 4.**
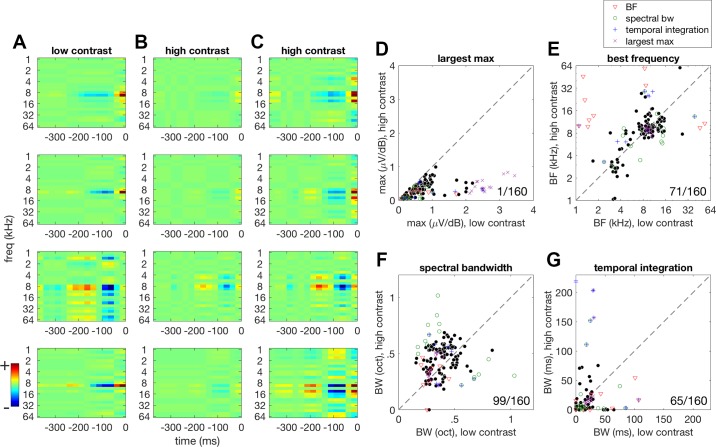
*A*: example spectrotemporal receptive fields (STRFs) estimated under low-contrast stimulation for 4 multiunits (MUs). Red areas of the STRF indicate spectrotemporal features of the stimulus that increase the amplitude of the MUA while blue areas indicate features that suppress multiunit activity (MUA). *Time 0* to 25 ms in the STRF corresponds to the first time bin in the history dimension. *B*: STRFs estimated under high-contrast stimulation for the same MUs revealing a strong suppression of the STRF coefficients. Color scaling is the same as in *A*. *C*: high-contrast STRFs, as shown in *B*, but with independent color scaling, revealing that spectrotemporal selectivity is broadly similar between contrast conditions (comparing low-contrast STRFs in *A* with high-contrast STRFs in *C*. *D*: by far the clearest effect of altering contrast is a reduction in stimulus sensitivity under high-contrast stimulation, as indicated by a reduction in the largest STRF coefficient (sign-rank test, *P* < 0.001). *E*: scatter plot showing the effect of contrast on best frequency (BF). The BF of each MU did not vary systematically with stimulus contrast (sign-rank test, *P* = 0.967). *F*: the bandwidth (BW) of tuning broadens slightly as stimulus contrast increases (sign-rank test, *P* < 0.001). *G*: temporal integration time showed no systematic changes between contrast conditions (sign-rank test, *P* = 0.054). For each parameter, outliers were identified as values that fell outside 1.96 SD of the distribution of these values. They are shown here in each plot using different markers and colors as indicated in the *inset*. The outlier parameters generally did not come from the same population of MUs, as indicated by the lack of overlap in outliers identified from the different parameters. Outliers were not excluded from statistical tests. Ratios in the corner of each plot indicate the number of MUs above the identity line, *y* = *x*, over the total number of MUs (*n* = 160).

We estimated STRFs for all MUs that we recorded in the mouse auditory cortex and included only those which had a predictive STRF (160/815). We estimated a separate STRF for each contrast condition, yielding separate high- and low-contrast STRFs (Fig. 4, *A*–*C*). This made it possible to assess whether changes in stimulus contrast have systematic effects on tuning.

The most striking change that occurred between STRFs from each condition was consistent with a gain change rather than a tuning change: a suppressive effect on responses during high-contrast stimulation. Of the MUs studied, 159 of the 160 showed a reduction in the largest STRF coefficient under high- compared with low-contrast stimulation (medians: low contrast: 0.6 µV/dB and high contrast: 0.29 µV/dB; sign-rank test, *P* < 0.001; Fig. 4*D*). The BF of the STRFs, defined as the frequency associated with the peak coefficient in the frequency kernel, did not change systematically with stimulus contrast (medians: low contrast: 9.5 kHz and high contrast: 9.5 kHz, sign-rank test, *P* = 0.967; Fig. 4*E*). Spectral bandwidth showed a small but significant increase during high-contrast stimulation (medians: low contrast: 1.45 oct and high contrast: 1.82 oct; sign-rank test, *P* < 0.001) (Fig. 4*F*), while no systematic changes in the temporal integration time of STRFs were found (medians: low contrast; 8.11 ms and high contrast; 5.01 ms; sign-rank test, *P* = 0.054; Fig. 4*G*).

The effects of contrast on STRF tuning were far less consistent than the suppressive effect on the largest STRF coefficient. Individual MUs exhibited changes in individual STRF parameters between conditions. This is likely to result in part from errors in estimating STRF coefficients. This problem is exacerbated in the present analysis because two STRFs were estimated for each MU, each using only half the data. A minority of MUs showed large changes in individual STRF parameters. It is possible that the MUs showing the greatest changes in BF, spectral bandwidth, and integration time across contrast conditions may simply not be well approximated by STRF models and, as a result, gave highly variable estimates of STRF parameter values. To assess whether such outliers come from a single subpopulation of MUs with poor STRFs, we identified outliers for each of these parameters independently and examined the overlap with these outliers. If the outliers for each parameter come from a single population, the MUs defined as outliers based on the different parameters should be largely overlapping. Outliers were defined as MUs that showed parameter changes between contrast conditions that fell outside 1.96 SD from the mean of the distribution of changes, as these values would have a <5% probability of occurring in such a distribution (i.e., *P* < 0.05). BF outliers are indicated by the inverted red triangle symbol, spectral bandwidth outliers are indicated by the green circle, and integration time outliers are indicated by the blue plus symbol, while largest max coefficient outliers are indicated by the purple cross (Fig. 4, *D*–*G*). Only 3 out of 50 outlier MUs were identified as outliers based on changes in more than one parameter, suggesting that these 50 outlier responses do not come from a single population that is uniformly poorly captured by STRFs.

**Fig. 5.**
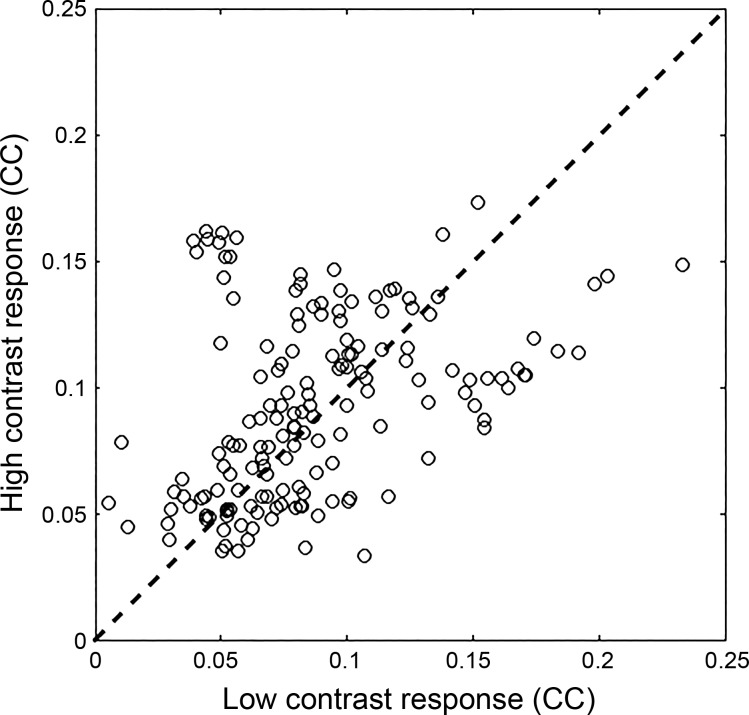
When a single spectrotemporal receptive field (STRF) fitted to both high- and low-contrast response data was used to predict responses, no significant difference was observed between high- and low-contrast conditions (CC) (sign-rank test, *P* = 0.139). This indicates that, despite small changes in tuning, a single STRF can be used to model contrast-dependent gain changes for these multiunits (*n* = 160).

We next examined whether a single STRF can be used to model responses to both high- and low-contrast stimuli without bias toward either condition. We investigated this by fitting a single STRF to 80% of both high- and low-contrast data and then using this STRF to predict the remaining 10% from each contrast condition separately. These STRFs predicted low- and high-contrast responses equally well (medians: high-contrast predicted: 0.09 and low-contrast predicted: 0.08; sign-rank test, *P* = 0.139; Fig. 5).

Overall, these results suggest that, although the estimated tuning parameters of individual MUs can change somewhat between contrast conditions, these changes can largely be explained by difficulties in estimating STRFs using only half of the available data. Since these changes were not systematic across the population of MUs, and STRFs were not significantly biased toward either contrast condition, it is reasonable to use a single STRF (plus a gain change) to describe neural responses across contrast conditions.

**Fig. 6.**
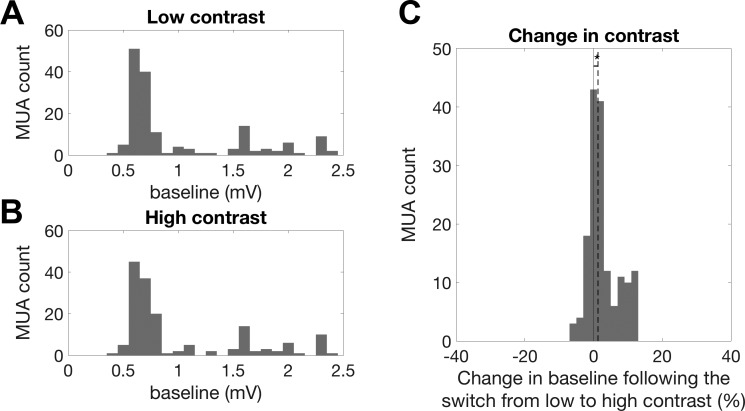
Changes in baseline activity between contrast conditions. *A*: histogram of baseline activity values across all multiunits (MUs) during low-contrast stimulation. MUA, multiunit activity. *B*: histogram of baseline activity values across all MUs during high-contrast stimulation. *C*: histogram of the %change in baseline activity under high- compared with low-contrast stimulation. Positive values indicate an increase in baseline activity during high-contrast stimulation. The solid line indicates 0% change. Across the population, MUs showed a small but significant increase in median baseline activity during high-contrast stimulation (sign-rank test, *P* < 0.001); *n* = 160).

**Fig. 7.**
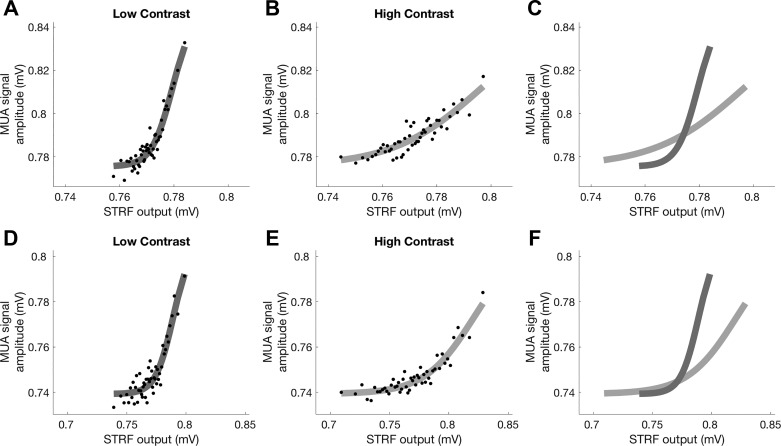
To quantify gain changes, a single spectrotemporal receptive field (STRF) was fitted to the data from each contrast condition for each multiunit (MU). An output nonlinearity that varied between contrast conditions was fitted to the output of this STRF. *A*: sigmoidal output nonlinearity fitted under low-contrast stimulation. The dots are binned responses to low-contrast stimulation. MUA, multiunit activity. *B*: nonlinearity fitted under high-contrast stimulation for the same MUs. The dots are binned responses to high-contrast stimulation. *C*: overlay of nonlinearities for both conditions. *D*–*F*: same as in *A*–*C* but for a second MU. The main effect of increasing contrast is to reduce the slope of this nonlinearity, capturing a change in the gain of the MUA.

We next measured the effect of stimulus contrast on neuronal gain and thresholds by incorporating data from both the high- and the low-contrast conditions into a single model. This model consisted of a single linear STRF, fitted to the data from both conditions (see Fig. 7). We then fitted a sigmoidal output nonlinearity (*Eq. 4*) that could vary in two parameters between contrast conditions, relating the output of the STRF to the real neuronal responses. Each sigmoid had four parameters that define *1*) the *y*-axis offset, *2*) *y*-axis range, *3*) *x*-axis offset, and *4*) the slope of the curve. The *y*-axis offset (*1*) represents “baseline activity” and reflects the additive effects of a constant electrical noise floor plus spontaneous, nonstimulus driven neural activity in the 300- to 6,000-Hz frequency band. Across the population, a very small but statistically significant increase in baseline activity was observed under high-contrast stimulation (median increase: 1.29%; sign-rank test, *P* < 0.001; Fig. 6*C*). The *y*-axis range (*2*) parameterizes the maximal range of firing of the MU. The *x*-axis offset (*3*) reflects the threshold while the slope of the curve (*4*) reflects the gain.

While *parameters 3* and *4* were allowed to vary between conditions to quantify the effects of changes in stimulus contrast, *parameters 1* and *2* were held constant. Although we observed differences in baseline activity as a function of stimulus contrast, these differences were negligibly small (<2%) and were therefore ignored. As was also the case in previous studies (Rabinowitz et al. 2011), neural responses were often not driven to saturation by the stimuli used (compare Fig. 7), making *parameter 2* poorly constrained by the data. However, to achieve our objective of measuring changes in neural gain, only the slope *parameter 4* needs to be measured accurately and that parameter is well constrained by the data. Consequently, whenever the responses fail to saturate over the range of observations in the data set, the saturation *parameter 2* simply becomes a free parameter of no importance. Thus, in line with much previous work, we fitted all data with sigmoidal output nonlinearities as just described, and we held *parameters 2* and *3* constant between contrast conditions and used contrast-related changes in *parameter 4* to quantify changes in neural gain.

**Fig. 8.**
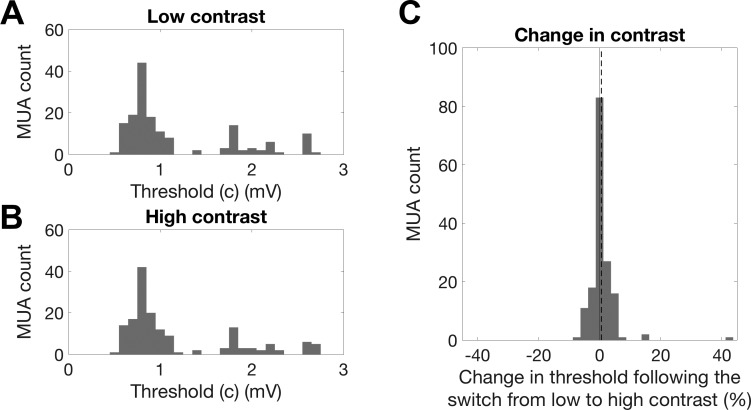
Changes in the output nonlinearity threshold parameter (c) between contrast conditions. *A*: histogram of threshold values across all multiunits (MUs) during low-contrast stimulation. MUA, multiunit activity. *B*: histogram of threshold values across all MUs during high-contrast stimulation. *C*: histogram of the %change in threshold under high- compared with low-contrast stimulation. Negative values indicate a reduction in threshold. The solid line indicates 0% change. MUs showed a small but significant increase in threshold during high-contrast stimulation (sign-rank test, *P* < 0.001; *n* = 160).

**Fig. 9.**
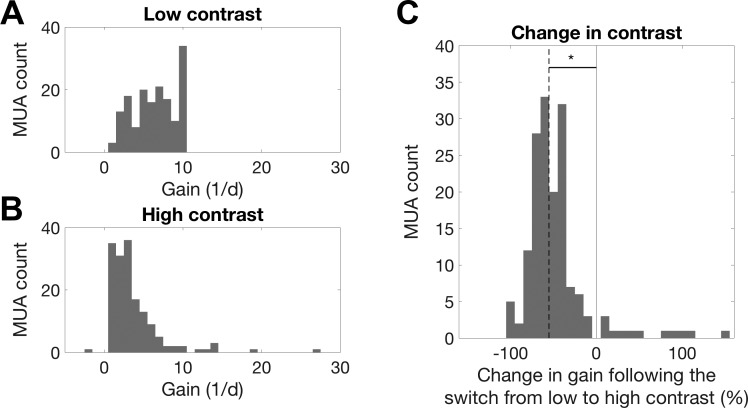
Changes in the output nonlinearity gain parameter (1/d) between contrast conditions. *A*: histogram of gain values across all multiunits (MUs) during low-contrast stimulation. MUA, multiunit activity. *B*: histogram of gain values across all MUs during high-contrast stimulation. *C*: histogram of the %change in gain under high- compared with low-contrast stimulation. Negative values indicate a reduction in gain, a leftward shift along the *x*-axis. The solid line indicates 0% change. The vast majority of MUs showed a compensatory reduction in gain during high-contrast stimulation, resulting in a significant decrease in gain across the population (sign-rank test, *P* < 0.001) (*n* = 160).

There was a small but significant increase in neuronal response thresholds during high-contrast stimulation (Fig. 8; median threshold change: 0.6%; sign-rank test, *P* < 0.001). Neuronal output gain exhibited sizable and statistically significant changes as a function of contrast conditions (Fig. 9). For the overwhelming majority of MUs, the gain decreased when contrast increased, indicating that gain changes tended to compensate for changes in stimulus contrast. Across the population, the median gain change was −55.2% (sign-rank test, *P* < <0.001; Fig. 9*C*), indicating that, in many units, gain changes completely compensated for the twofold increase in stimulus contrast. After outliers that were >3 SD away from the mean were removed, contrast-dependent changes in gain were not significantly correlated with changes in threshold (*R* = −0.08, *P* = 0.35).

#### Gain control is strongest in deep cortical layers.

**Fig. 10.**
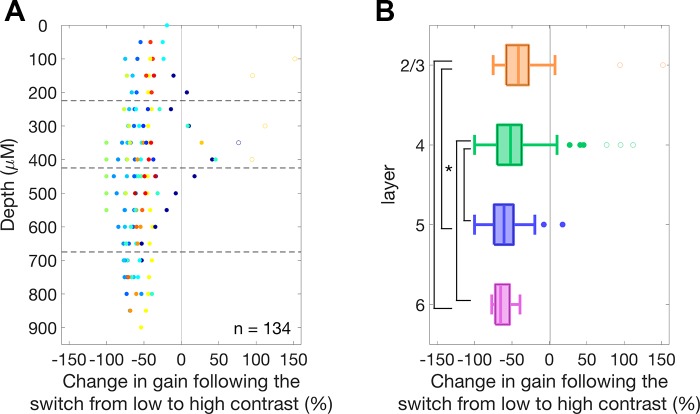
*A*: strength of gain change for each multiunit (MU) plotted as a function of cortical depth from the layer 1–2/3 border. Putative layer boundaries are indicated by lines and data points are color-coded by penetration. *B*: box plots of gain control within each cortical layer. The median gain change was significantly greater (*P* < 0.05) in layers 5 and 6 compared with layer 2/3 and layer 4, as indicated by the asterisk. Outliers were excluded from the analysis and are indicated here by open circles.

Previous investigations of CGC in the auditory cortex did not investigate possible systematic differences according to the cortical layers in which the recordings were made (Rabinowitz et al. 2011). As described previously, we used linear probes that allowed us to identify cortical layers using CSD analysis (Fig. 3). A reversal in the CSD profile is observed at the border of layer 1 and 2/3, and this feature was used to align recordings from different penetrations. After alignment of our recordings, MUs were assigned to cortical layers based on their cortical depth from the layer 1–2/3 border (Fig. 10*A*), using experimentally obtained measurements of layer thickness (Anderson et al. 2009; Ji et al. 2016). Layer estimates were inspected visually for features such as an early current sink in layer 6 and were compared with the depth distribution of spiking responses to confirm layer estimates. Linear probes were used in 19 of the 24 experiments, making it possible to estimate cortical depth for 139 of 160 MUs.

To test whether the strength of gain control was significantly different between layers, a two-factor ANOVA was performed with strength of gain control as the response variable, layer (2/3, 4, 5, and 6; df = 3) as one factor, and penetration as the other (df = 16). Five outlier MUs, defined as showing gain changes >3 SD from the mean, were observed in layers 2/3 and 4 and were excluded from the analysis. The majority of these outliers had poor STRF fits, which may account for their outlier status. The layer means were layer 2/3: −41.6%, *n* = 21; layer 4: −54.2%, *n* = 39; layer 5: −60.6%, *n* = 50; and layer 6: −65.1%, *n* = 24. Both factors (layer and penetrations) were found to be significant (*P* < 0.001 in both cases). Pairwise significance tests between the layer means (Tukey-Kramer corrected for multiple comparisons) found that there were significant differences (at *P* < 0.01) in the strength of gain control between layers 2/3 and 5 (*P* = 0.009) and between layers 2/3 and 6 (*P* = 0.008). Similarly there were significant differences in the strength of gain control between layers 4 and 5 (*P* = 0.007) and between layers 4 and 6 (*P* = 0.0096). Other differences were not significant (*P* > >0.05).

## DISCUSSION

Here we characterized CGC in the mouse to test whether it resembles that seen in other species, as would be expected if this was a “canonical” computation, and to lay a foundation for investigations into the mechanisms underpinning it. Mice were anesthetized for these experiments as this permitted more precise control over stimulus presentation and previous work in the ferret reported no systematic difference between contrast gain control in anesthetized and awake preparations (Rabinowitz et al. 2011). Previous work in the mouse auditory cortex has also shown that stimulus-driven response properties are robust across anesthetic states (Guo et al. 2012). Whether anesthetics have laminar-specific effects in the rodent sensory cortex is not known, although the anesthetized preparation has previously been used to examine the laminar variation of response properties in auditory cortex (Sakata and Harris 2009; Szymanski et al. 2011).

We first investigated the effects of stimulus contrast on the spectrotemporal tuning of MU responses in the mouse auditory cortex. If cortical responses show a pure gain change in response to a change in stimulus contrast, tuning properties should remain the same. In the ferret, 89% of MUs maintained their best frequency within 1/6 of an octave when contrast was changed by a factor of 3 (Rabinowitz et al. 2011). In the mouse, however, only 64% of MUs with predictive STRFs maintained their best frequency, within 1/2 an octave when contrast was changed by a factor of 2, indicating that a greater proportion of units showed changes in BF with stimulus contrast. As in the ferret, however, there was no systematic effect of stimulus contrast on BF (Rabinowitz et al. 2011).

In the ferret, a threefold increase in contrast was found to result in slightly narrower bandwidth of tuning under high-contrast stimulation (Rabinowitz et al. 2011). We found that, in the mouse, the opposite pattern occurs. For an approximately twofold increase in contrast, there is a small but significant tendency for STRFs to increase their bandwidth as contrast increases. The general structure of STRFs is conserved between mouse and the ferret, as well as across anesthetic states (Rabinowitz et al. 2011), although interspecies differences in receptive field structure could in theory account for these differences. Spectral bandwidths in the ferret ranged from ~0.25 octaves to ~1.25 octaves, while in the mouse bandwidths ranged from ~0.1 to ~1 octave. However, differences in methodology make precise comparisons in tuning parameters across species difficult to make. DRCs used here consisted of 25 pure tone components covering 6 octaves (1–64 kHz) spaced 1/4 octave apart. In the ferret, DRCs comprised 34 pure tones and covered 5.5 octaves (500 Hz to 22.6 kHz) spaced 1/4 octave apart (Rabinowitz et al. 2011). This could result in differences in measures such as tuning bandwidth between these experiments. As in the ferret (Rabinowitz et al. 2011), no systematic changes in the temporal tuning of STRFs were observed and the most striking change that occurred between STRFs from each condition was a suppressive effect on the STRF coefficients under high-contrast stimulation. Every MU recorded apart from one showed a reduction in the largest STRF coefficient under high-contrast compared with low-contrast stimulation. This suggests that the effects on bandwidth may be functionally less important compared with a possible gain change in response to the change in contrast. We cannot rule out the possibility, however, that there are tuning shifts in individual units, or that there may be subtle population-wide effects that our analysis cannot detect.

These results indicate that, as in the ferret, neuronal responses in the mouse auditory cortex undergo a reduction in gain in response to an increase in stimulus contrast. We quantified any contrast-dependent changes in gain by fitting a single STRF for each MU with an output nonlinearity that could vary between contrast conditions. This allowed us to estimate the effect of contrast on gain and response thresholds for each MU. To justify the use of a single STRF across both contrast conditions, we show that the combined STRFs we estimate provide a fair description of neural responses in each of the two conditions separately (Fig. 4). This was necessary to rule out the possibility that the STRFs were biased in such a way that they mainly reflected the responses in the high-contrast condition, and provided only a poor fit to the low-contrast data. We found that 92.5% of MUs in the mouse auditory cortex showed a reduction in gain in response to high-contrast compared with low-contrast stimulation. In the ferret, a contrast-dependent increase in threshold that correlated with the strength of gain control was also observed (Rabinowitz et al. 2011). A small but significant increase in threshold was also observed here. The median decrease in gain across the population was 55.2% in response to an approximate doubling of stimulus contrast, indicating that in the mouse auditory cortex contrast gain control may be completely compensatory. This was not found to be the case in ferret auditory cortex, where a threefold increase in contrast was found to produce a 50% reduction in gain, instead of the 66% reduction that would be entirely compensatory (Rabinowitz et al. 2011). These findings indicate that unlike ferret auditory cortex, CGC in the mouse auditory cortex may approximate complete normalization, as is thought to be the case in V1 (Heeger 1992).

Contrast does not have a single universally accepted definition but typically refers to the range of values that a relevant stimulus parameter takes. Here we use the variance of the sound-level distribution operationally to quantify contrast. While changes in gain compensate for changes in the variance of the sound-level distribution, our data cannot address what effects changes in other properties of the input distribution, such as the kurtosis or skewness, may have on neural responses. That is, neurons may be sensitive to other statistical moments of the input distribution. Changes in mean sound level are known to result in compensatory adjustments in the offset of the dynamic range of neurons in the auditory nerve (Wen et al. 2009) and inferior colliculus (Dean et al. 2005). For this reason we kept the mean sound-level constant to examine the effect of stimulus contrast. However, higher-order moments of the input distribution may also produce compensatory adjustments in neural responses throughout the auditory system. We did not examine whether spectral and temporal contrast have independent effects on neural responses, although stimuli that vary in either spectral (Barbour and Wang 2003) or temporal contrast (Schreiner and Urbas 1986) have been developed, which could be used to probe these components independently.

The finding that CGC is prevalent in the mouse auditory cortex allows for investigations into the cellular mechanisms underlying this computation. Great progress has been made in understanding the mechanisms underlying CGC in themouse visual cortex using optogenetics, where PV-expressing interneurons have been implicated (Atallah et al. 2012; Wilson et al. 2012). A similar line of investigation in the mouse auditory cortex will not only increase our understanding of information processing mechanisms in the auditory system but will also allow for direct comparisons with findings in visual and other sensory modalities, enabling the question of whether a canonical gain control mechanism exists to be addressed experimentally. For example, differences in PV interneuron tuning have been observed between visual (Kuhlman et al. 2011; Zariwala et al. 2011) and auditory cortexes (Moore and Wehr 2013), but the manner in which these differences relate to the computations being performed by the cortical populations remains unknown. The findings presented here indicate that, while CGC is present in the mouse auditory cortex, interesting differences between species and modalities may exist. This may be seen as evidence for a “serial homology” hypothesis of functional cortical organization (Harris and Shepherd 2015), in which different cortical areas employ modified versions of common computations and mechanisms that are tailored to the system in question.

As a first step toward understanding the circuit basis of CGC in the mouse auditory cortex, we tested predictions made from different circuit hypotheses regarding the laminar organization of CGC. We found that the strength of CGC exhibits modest, but significant, laminar variation in the mouse auditory cortex, with CGC being strongest in layers 5 and 6. It is not known whether similar laminar variation in contrast gain control is found in other sensory cortical areas. In V1, thalamic input has been shown to drive corticothalamic projecting layer 6 neurons, which in turn drive a translaminar projecting PV interneuron subtype via facilitating synapses (Bortone et al. 2014; Olsen et al. 2012). Inhibition via this route has been found to reduce gain across all other cortical layers in V1, but not in layer 6. The presence of strong CGC in layer 6 of the auditory cortex indicates that it may not be implemented through translaminar projecting layer 6 PV interneurons (Bortone et al. 2014; Olsen et al. 2012), as this mechanism would require layer 6 responses to be invariant to stimulus contrast. Layer 6 corticothalamic projecting neurons may contribute to CGC, however, via interactions with the thalamus. Activation of corticothalamic projecting neurons found in layer 6 of auditory cortex has been found to produce changes in gain in both the MGB and auditory cortex (Guo et al. 2017). The increased strength of contrast gain control in deep cortical layers may therefore reflect the involvement of these neurons in a corticothalamic circuit for gain control. Simultaneous recordings in these two structures combined with the measurements of the temporal evolution of gain control would be necessary to investigate this possibility.

The presence of strong CGC in layer 4 is consistent with gain control being primarily implemented in, or inherited by, this layer as it is thought to represent the earliest stage of cortical processing (Douglas and Martin 1991). However, the strength of CGC showed variation across the layers of the auditory cortex. This indicates that intracortical mechanisms in auditory cortex may also contribute to CGC following the initial processing of sensory information in layer 4. Thalamic inputs to auditory cortex have been found to specifically innervate PV interneurons in all cortical layers (Ji et al. 2016), raising the alternative possibility that feedforward inhibition across the cortical column may be the primary basis for CGC, rather than it being simply inherited from layer 4. Circuit-level experiments, made possible by the work presented here, will be necessary to test these models experimentally.

## GRANTS

This work was supported by the Wellcome Trust through Principal Research Fellowship Grants WT076508AIA and WT108369/Z/2015/Z (to A. J. King) and a 4-yr studentship (096588/Z/11/Z; to J. E. Cooke).

## DISCLOSURES

No conflicts of interest, financial or otherwise, are declared by the authors.

## AUTHOR CONTRIBUTIONS

J.E.C., A.J.K., B.D.W., and J.W.H.S. conceived and designed research; J.E.C. performed experiments; J.E.C., B.D.W., and J.W.H.S. analyzed data; J.E.C., A.J.K., B.D.W., and J.W.H.S. interpreted results of experiments; J.E.C. prepared figures; J.E.C. drafted manuscript; J.E.C., A.J.K., B.D.W., and J.W.H.S. edited and revised manuscript; J.E.C., A.J.K., B.D.W., and J.W.H.S. approved final version of manuscript.
